# Predicting the rental value of houses in household surveys in Tanzania, Uganda and Malawi: Evaluations of hedonic pricing and machine learning approaches

**DOI:** 10.1371/journal.pone.0244953

**Published:** 2021-02-11

**Authors:** Weldensie T. Embaye, Yacob Abrehe Zereyesus, Bowen Chen

**Affiliations:** 1 Department of Agricultural Economics, Kansas State University, Manhattan, Kansas, United State of America; 2 International Trade and Development Branch, Markets and Trade Economics Division, U.S. Department of Agriculture, Economic Research Service, Kansas City, MO, United State of America; 3 Department of Agricultural and Consumer Economics, University of Illinois, Urbana, IL, United State of America; The Bucharest University of Economic Studies, ROMANIA

## Abstract

Housing value is a major component of the aggregate expenditure used in the analyses of welfare status of households in the development economics literature. Therefore, an accurate estimation of housing services is important to obtain the value of housing in household surveys. Data show that a significant proportion of households in a typical Living Standard Measurement Survey (LSMS), adopted by the Word Bank and others, are self-owned. The standard approach to predict the housing value for such surveys is based on the rental cost of the house. A hedonic pricing applying an Ordinary Least Squares (OLS) method is normally used to predict rental values. The literature shows that Machine Learning (ML) methods, shown to uncover generalizable patterns based on a given data, have better predictive power over OLS applied in other valuation exercises. We examined whether or not a class of ML methods (e.g. Ridge, LASSO, Tree, Bagging, Random Forest, and Boosting) provided superior prediction of rental value of housing over OLS methods accounting for spatial autocorrelations using household level survey data from Uganda, Tanzania, and Malawi, across multiple years. Our results showed that the Machine Learning methods (Boosting, Bagging, Forest, Ridge and LASSO) are the best models in predicting house values using out-of-sample data set for all the countries and all the years. On the other hand, Tree regression underperformed relative to the various OLS models, over the same data sets. With the availability of abundant data and better computing power, ML methods provide viable alternative to predicting housing values in household surveys.

## Introduction

According to World Bank [1], the number of world population in poverty had declined from 1.85 billion in 1990 to 767 million in 2013. The World Bank’s Living Standards Measurement Study-Integrated Surveys on Agriculture (LSMS-ISA)is a typical household survey conducted in many countries in Sub-Saharan Africa and elsewhere [2].The LSMS program objectives are to improve the quality of household survey data, increase the capacity of statistical institutes to perform household surveys, improve the ability of statistical institutes to analyze household survey data for policy needs, provide policy makers with data that can be used to understand the determinants of observed social and economic outcomes [2]. Based on the concept of money metric utility as a theoretical foundation [3], consumption expenditure is believed to be appropriate for measuring welfare in households. In many developing countries, gathering household income data that show greater variability over time is expensive and difficult [4]. Aggregate household expenditure tends to show less variability (or volatility), as compared to household’s income. Deaton and Zaidi [4] state that annual expenditure that does not change much over time can be estimated at cheaper cost through surveying two weeks of households’ consumption expenditure than household’s income. Based on data such as the LSMS-ISA, aggregate expenditure is sub-aggregated in to food, non-food, durable goods, and housing categories for purposes of welfare analyses.

**Fig 1:**
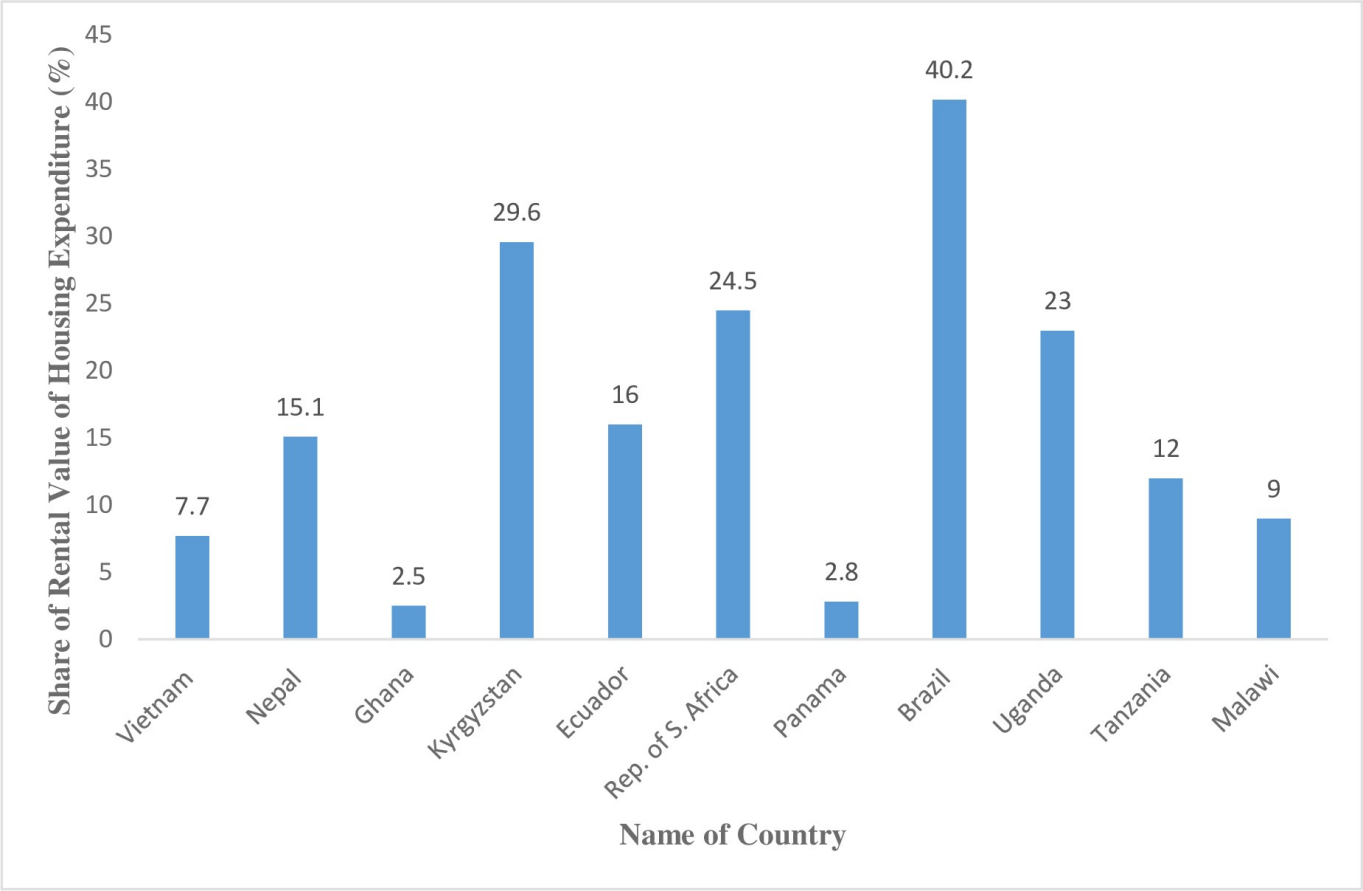
Percentage share of rental value of housing on overall household expenditure for different countries. Authors data set, and Deaton and Zaidi [4].

The value of house is a major component of the household’s aggregate expenditure. The share of the value of house as a percentage of the aggregate expenditure varies by countries as shown in Fig 1. Although these numbers are from different countries across different periods, these numbers support the fact that the value of house represents a significant portion of the household’s aggregate expenditure. The highest share is observed in Brazil (40%) followed by Kyrgyzstan (29.6%). The lowest shares are reported in Panama (2.8%) and Ghana (2.5%). It is expected that the figures might change when disaggregated by regions and districts. For example, Zereyesus et al. [5], indicate that in northern Ghana, following food expenditure, house rent was ranked as the second highest category, forming 16% of household’s aggregate expenditure.

Data show that a significant proportion of house ownership in a typical household survey is self-owned. Deaton and Zaidi [4] observed that of all the household consumption aggregate, the housing sub-aggregate is often one of the most problematic. Implicit rental values (i.e. values obtained by asking how much households would have paid if they were renting the house) and hedonic pricing (using econometric model to predict house values) are common approaches used to estimate the value for self-owned housing service in the World Banks’s Living Standards Measurement Studies [4, 6, 7].

In hedonic pricing approach, the rental value of a house is modelled as a function of house characteristics. The house characteristics are regressed on house rental values using the subpopulation of house renters. Using these parameterizations, the value of self-owned house rent is predicted. Hedonic pricing is often predicted using an Ordinary Least Squares (OLS) method with and without accounting for spatial autocorrelation effects (see [4, 8, 9–11]).

Machine Learning (ML) approaches may provide better out-of-sample predictive values considering higher dimensionality of data structures. Vinod [12] noted that experiments consistently supported a better prediction error by ML methods over OLS. Machine Learning approaches are widely used in computer science field and have recently been adopted in the statistics and economics fields [13–16]. Limsombunchai et al. [17] used OLS and ML method (Artificial Neural Network) to predict the value of owning a house in New Zealand and found that ML method was better in the prediction. Similarly, Do and Grudnitski [18] applied OLS and Artificial Neural Network approach to predict the selling price of single-family homes in San Diego, California, the United States of America. Their results indicated that the ML method outperformed the OLS regressions in predicting the housing values. Picchetti [19] also applied ML approach (Gradient Tree Boosting Algorithm) to estimate the residential property prices and found that ML methods resulted in better out-of-sample predictions compared to the OLS method. Recently, Yang [20] assessed the effect of fracking on housing prices using both hedonic and machine learning (Random forest and Boosting) approaches and concluded that ML methods can significantly improve prediction accuracy.

In this article, we aim to empirically examine Machine Learning (ML) approaches, shown to uncover generalizable patterns [14] based on a given data, in reference to hedonic pricing approach, to obtain a better overall prediction of rental value of house using multi-period household-level survey data from three sub-Saharan Africa countries (Uganda, Tanzania, and Malawi). The ML approaches evaluated are Ridge, LASSO, Tree, Random Forest, Bagging, and Boosting. The hedonic pricing approach is implemented using a standard Ordinary Least Squared (OLS) regression method. Recent developments in spatial autocorrelation analyses (SEM and SAR) are applied to implement the OLS to account for spatial autocorrelation issues [9–11]. As Athey [16] emphasizes, the goal is to predict, not to forecast, rental values of houses in an independent dataset based on the realized values of household features. In our case, the independent dataset would be the sample of non-rented households in the respective household survey data for the given year of data used from Uganda, Tanzania, and Malawi.

Besides, results from the study show that both the ML approaches and the hedonic approach identified similar variables as determinants of rental values: the number of rooms, availability of electricity, private tap water, and toilet characteristics. Judged by the Mean Squared Error (MSE) performance, Random Forest, Boosting and Bagging regression outperformed in-sample prediction performances of all other ML and OLS approaches for all the data sets. The OLS predictions outperformed Ridge, LASSO, and Tree regressions countries and years. Boosting is the best model in predicting house rental values using out-of-sample data set for all the countries and all the years. Bagging, Random Forest, Ridge and LASSO also outperformed OLS in out of sample prediction. Tree regression is the least performer in the group.

The rest of the study is organized in the following manner. The next section provides an account of the methodology used for the study providing brief review of the theoretical background of the hedonic pricing approach and the Machine Learning approaches. The section on data documents the data used and provides descriptive analyses of the main variables used for the estimations. The results and discussion section presents empirical estimation results and discusses the performances of the hedonic approaches and ML approaches for in-sample and out-of-sample predictions. The last section wraps up the study with the main findings and conclusions and pointing for possible extensions of the current study.

## Methodology

In his introductory note to the many of the novel machine learning tools, Varian [13] classified data analysis in statistics and econometrics into 1) prediction, 2) summarization, 3) estimation, and 4) hypothesis testing. The primary concern of ML is prediction. In ML, the focus is to find some function that provides a good prediction of Y as a function of X. The vector X is referred to as ‘predictors’ or ‘features’. In general, these ML methods deal with high-dimensional data by shrinking some of the variables to zero and retaining the most important covariates. Machine Learning methods perform better on modeling data with dimensionality problems such as serial correlation [21, 22]; spatial correlation [23, 24]; small sample size problem [25, 26]; or a combination of all [27].

A practical concern that generally motivates the adoption of ML procedures is the potential for severe overfitting in high-dimensional settings. To avoid over-fitting, most ML procedures for “supervised learning” (that is, regression and classification methods used for prediction: prediction of Y as a function of X) involve two key features, (i) regularized estimation and (ii) data-driven choice of regularization parameters [28]. Varian [13] stated that ML overcomes overfitting complexity of models using three ways: (1) regularization, penalizing for some parameters to gain model simplicity, which in turn are more effective in prediction than complex models; (2) dividing the data into training and testing, where training is used to fit the model, while testing is considered as out-of-sample and used to evaluate performance of the model; and (3) using a tuning measure that produces a best of out-sample prediction. K-fold cross validation, which we elaborate later, is the most common and standard way to estimate the tuning parameter.

Following is a brief review of the hedonic pricing approach and the various ML approaches.

### Hedonic pricing approach

The hedonic pricing model has extensively been used to estimate the value of a commodity including housing, properties, and agricultural crops [17, 29, 30] or to value environmental quality, primarily to value environmental disamenities in urban areas including air pollution and proximity to hazardous waste sites [31, 32]. Sirmans, Macpherson, and Zeitz [33] provide a summary of the underlying theory and empirical applications of the hedonic pricing models. The model is based on establishing a functional relationship between the value of housing and individual attributes that comprise the house [33]. House attributes may include such characteristics of a house as number of bedrooms, number of bathrooms, number of fireplaces, parking facilities, living area and lot size that are implicitly embodied in goods and their observed market prices [17, 34–37]. Consumers purchase goods that represent the bundles of attributes that maximize their underlying utility functions [38].

The hedonic pricing model decomposes the house price into characteristics such as interior, exterior and others that have a bearing on the house sales price [39]. For the current study, the rental value of a house is modelled as a function of housing characteristics [40]. The housing characteristics are regressed on house rental values using the subpopulation of house renters. Using these parameterizations, the value of self-owned house rent could then be predicted.

The hedonic price model, often implemented using an Ordinary Least Squares (OLS) regression approach, takes the following general form:

$$Y=\beta^{\prime }X+\varepsilon ,\;\varepsilon ∼(0,\sigma^{2})$$ (1)

Where *Y* is the outcome variable (house rental value) in its natural log, *X* represents a vector of explanatory variables, *ε* represents an nx1identically and independently distributed (IID) error term, and *β* refers to the vector of parameters. The regression model is used to find the optimal coefficient estimates that give the least squared errors.

Spatial correlation [23, 24] is an important consideration while modeling housing values. Eq 1 could be extended in two ways to account for spatial effects [9]. First, various authors develop a spatial autoregressive process represented by a spatial lag model (SAR) as follows [9–11]:

$$Y={\rho WY+\beta }^{\prime }X+\varepsilon ,\;\varepsilon ∼(0,\sigma^{2})$$

where ρ is the spatial autoregressive coefficient, W is a square spatial weights matrix which captures the definition of “neighborhood” chosen, and *β*′*X* and ε are as defined before. This W contains the spatial relations among observations and controls for the impact of any house rental value on neighboring house rental values across space. Second, the data generation process can be represented by the spatial error model (SEM) if the autocorrelation occurs because some omitted causal factors in the hedonic price function exhibit spatial autocorrelation in the disturbance term ε. This implies that house rental price at any location in Eq (1) may also be explained by the omitted variables at neighboring housing observations such that the error term in Eq 1 is specified as *ε* = *λWε*+*u*, where λ is the coefficient on the spatially correlated error structure and u is nx1 vector of iid errors uncorrelated with the observed explanatory variables.

### Bias-variance tradeoff

Based on the OLS estimations of the variable coefficients, suppose that our fitted model is given by $\hat{Y}=\hat{\beta }X$. Relative to the actual values of *Y*, the Prediction Error (PE) at particular point of *X*_0_ is given by:

$${PE(X}_{0})=E_{Y/X=X_{0}}\{{\left(Y-\hat{Y}\right)}^{2}|X=X_{0}\}$$

After some manipulation of the above prediction error equation, the PE could be decomposed into the following parts as:

$${PE(X}_{0})={\sigma_{\epsilon }}^{2}+{Bias}^{2}\left(\hat{Y}(X_{0}\right))+Var(\hat{Y}(X_{0})).$$

This decomposition is referred to as the bias-variance tradeoff. As more terms are added to the model, the estimates are influenced by high variance. Hence, removing some variables from the model may add little bias in the model but it may reduce the variance significantly, which in turn reduces the PE. This bias-variance decomposition is at the core of ML models such as Ridge and LASSO, which attempt to introduce bias into the regression solution, but reduce the variance significantly as compared to the OLS solutions. While OLS regressions are known to provide unbiased coefficient estimates, the lower variance from such ML methods produces better Mean Squared Error (MSE) results.

As stated previously, ML models generally partition the data into ‘training’ and ‘testing’ parts. The training data are used to build the model; while the testing data are used to test the prediction power of the model. A K-fold cross-validation, an essential part of the tuning measure, is considered for model selection. During the cross-validation, the data set is divided randomly into K-sections of equal sizes. A model is fitted using one set and the parameters from the fitted model are used to predict for the rest, the K-1 section of the data. This process is executed K-times, i.e. as many as the number of folds. The MSE at each K-fold predictions are calculated, and the performance of the model is evaluated based on the average of the K-times the MSE. A model with minimum average MSE over K-fold predictions is considered as the best model for prediction. In other applications, Akaike Information Criteria (AIC), Bayesian Information Criteria (BIC), and other methods can also be used for model selection. The advantage of cross validation over BIC, AIC, and others is that it depends on fewer assumptions [13]. The detailed procedure on how K-fold cross-validation works can be found in James et al. [41].

### Machine learning approaches

The ML methods used in this article are Ridge regression, LASSO, Tree Regression, Bagging, Random Forest and Boosting.

#### Ridge regression

Ridge regression is an extension of the OLS in that it minimizes the MSE by applying a penalty parameter, which depends on the complexity of the model. Ridge model is specified as:

$$Minimize\;\sum_{i}{\left(Y_{i}-{\beta X}_{i}\right)}^{2}+\lambda \sum_{j}{\beta_{j}}^{2}$$ (2)

where *λ* represents the penalty parameter. When *λ* = 0, Ridge is equivalent to OLS.

#### Least absolute shrinkage and selection operator regression

Unlike in the Ridge, the Least Absolute Shrinkage and Selection Operator (LASSO) penalizes the summation of the absolute value of the coefficients in. The LASSO model is given by:

$$Minimize\;\sum_{i}{\left(Y_{i}-{\beta X}_{i}\right)}^{2}+\lambda \sum_{j}\left|\beta_{j}\right|$$ (3)

The LASSO reduces some of the coefficients to zero. Ridge is chosen over LASSO when many explanatory variables have small impact on the outcome variable. LASSO is chosen over Ridge when some explanatory variables have large effect on the outcome variable. We assign values to *λ*, and calculate the cross-validation errors at each value of *λ* [17, 28]. Mean Square Error is drawn against various values of *λ* and the model that provides minimum Mean Square Error is selected for the analysis.

#### Tree regression

Unlike the previous ML approaches, Tree Regression is a non-parametric approach that does not require specification of any particular functional form. It splits the data into subtrees based on the variable that best explains the data. It keeps dividing the data into subtrees until (1) there is single observation in each subtree, (2) all data in the subtree are identical, or (3) number of subtrees can be determined by the practitioner.

The model for the regression tree is given by:

$$Y=\sum_{n}\beta_{n}1(X\epsilon R_{n})$$ (4)

The indicator 1(*X*ϵ*R*_*n*_) is a dummy variable with a value of X = 1 if the variable is with subtree n, and 0 otherwise. The *β*_*n*_ is the mean value of the dependent variable of the data in subtree n. The mean value serves as the predicted value of the dependent variable from new explanatory variables. Tree Regression is applied when the response variable is continuous (e.g. rental value of house). However, when the response variable is discrete, then the model is called classification tree. Tree regression holds any number of variables [42]. In a linear regression model, the entire data set is represented using the same parameters. However, in Tree Regression, splitting the data set helps to fit the model using relatively homogenous data set. Data partitioned to relatively homogenous level avoids or reduces degree of collinearity and may potentially fit well when the response variables have non-linear or more complex relationship with the explanatory variables. Tree Regression may not perform well with small sample data. Moreover, linear model may perform well when the dependent variable has generally linear relationship with the explanatory variable. To overcome these potential challenges in Tree Regression, various tree families (Bagging, Random Forests, and Boosting) have been developed as discussed below.

#### Bagging

Bagging is another type of Tree Regression applied to reduce potentially high variance using a bootstrapping method [43]. Bagging arises to overcome the problem of high variance (due to small sample size) by Tree Regression. Generally, bootstrapping, a repetitive random sampling with replacement, reduces variance of the parameter of estimates [43]. Fitting the prediction model using two randomly partitioned training data sets may give diverse outcomes (high variance), which leads to lower quality predictions. To overcome this problem, Bagging, that potentially form large group of training data set for regression tree through random resampling with replacement yields low variance prediction results [41]. One of the drawbacks for Bagging is that it may not perform well when the predictive power of some of the Bagging Regressions are much better than others. Variables that are less important or weak covariates in the regression yields high variance during bootstrapping, which in turn leads to poor prediction. An ML method that comes to overcome this challenge is the Random Forest.

#### Random forests

Random Forest provides better predictions when there are highly correlated explanatory variables. Although Random Forest follows similar procedures as Bagging, it is possible that strong covariates could be chosen at random. The foundation of Random Forest rests on randomly picking a certain number of the explanatory variables at each sampling. A more detailed theoretical and practical application of Random Forest can be found in Liaw and Wiener [44] and Breiman [45]. Randomly selecting part of the predictors results in a much different outcome than Bagging, which uses the sample explanatory variables at each sampling. The underlining assumption is that average outcomes of unrelated results may bring larger variance reduction than averaging similar results. For example, James et al. [41] argued that averaging various uncorrelated quantities offer much less variance compared to highly correlated quantities. By picking only part of the available predictors, Random Forest helps to overcome overfitting problem due to a large number of covariates [45]. A drawback of Random Forest is that picking only relatively less strong covariates, but not strong explanatory variables, may lead to largely poor predictions.

#### Boosting

Unlike Random Forest, that build trees independent of each other, Boosting, another type of Tree Regression, builds the trees sequentially [28, 41]. Each previous tree has substantial effects on the construction of the following tree. Each tree is built using the information from the previous created tree. Later, a tree is fitted to the residuals, instead of the outcome variable of the former tree, then the errors are updated by adding the new decision tree to the fitted function. This continuous fitting of data over the error terms shrinks the variance of the model, which in turn results in good prediction [41]. This strategy avoids fitting a large single tree in to the data that potentially reduces the possibility of overfitting the data. Unlike the other tree families, Varian [13] indicated that Boosting is useful for any functional forms (linear, non-linear, logistic, etc.). Boosting is less sensitive to changes in training data set [44, 46]. It is important to point out here that heterogeneity of data may cause users to question the results of the regression. Freund and Schapire [46] caution that the fact that Boosting retains the properties of the previous regression, may unnecessarily dictate the behavior of the following fitting data set.

## Data

Data used in the article comes from the nationally representative Living Standards Measurement Study (LSMS) of household level surveys compiled by the World Bank collected in 2010/2012, 2014/2016, and 2014/2016, in Uganda, Tanzania, and Malawi, respectively. The use of data from these three countries is mainly based on the availability of the data set and the completeness of the variables used to achieve the objectives of the current study. Information from surveyed households pertaining to rental values and other variables of interest describing the location, quality of housing, and other features was extracted from the survey data for use in the current article. The number of houses that were rented out during the survey in each survey year, by country, form the basis of the sample sizes for the analyses. In Uganda, the sample sizes are 242 and 263 in the years 2010 and 2012, respectively. In Tanzania, the sample sizes are 873 and 1,363 in the years 2014 and 2016, respectively. In Malawi, the sample sizes are 662 and 1,479 in the years 2014 and 2016, respectively (Table 1). The rental monetary values from the domestic currency in each of the countries is converted to a United States Dollar to facilitate easier comparison and analyses of results. The year 2012 is taken as a base year and the rental value of each year is converted to the year 2012 prices. Districts provide some level of administrative subdivisions in the respective countries. There are 66 districts, 9 districts, and 32 districts included in the data from Uganda, Tanzania, and Malawi, respectively.

**Table 1:** Summary statistics of variables used in the house rental value predictions.

Variable	Definitions	Mean values (standard deviations)					
		Uganda		Tanzania		Malawi	
		2010	2012	2014	2016	2014	2016
Annual rent	Rent paid per month in dollar	22.18 (25.25)	23.46 (36.20)	17.89 (21.30)	22.65 (24.20)	20.34 (23.37)	17.39 (19.96)
Dwelling	1 if the room is located within a house, 0 otherwise	0.65 (0.46)	0.66 (0.46)	0.30 (0.40)	0.37 (0.48)	0.92 (0.28)	0.92 (0.27)
Roof	1 if roof type is mud, 0 otherwise	0.92 (0.27)	0.95 (0.26)	0.95 (0.25)	0.96 (0.25)	0.86 (0.35)	0.89 (0.31)
Floor	1 if floor is mud, 0 otherwise	0.68 (0.46)	0.34 (0.46)	0.22 (0.41)	0.91 (0.41)	0.80 (0.41)	0.77 (0.41)
External wall	1 if external wall is in mud, 0 otherwise	0.70 (0.45)	0.68 (0.45)	0.54 (0.45)	0.63 (0.48)	0.95 (0.21)	0.97 (0.18)
Number of rooms	Number of rooms rented	1.86 (1.12)	1.84 (1.16)	1.68 (0.91)	1.61 (0.92)	2.09 (0.97)	2.25 (0.98)
Electricity	1 if the house has electricity, 0 otherwise	0.35 (0.48)	0.37 (0.49)	0.53 (0.50)	0.59 (0.49)	0.42 (0.50)	0.44 (0.49)
**Water sources**							
Private tap water	1 if private tap water source, 0 otherwise	0.03 (0.16)	0.07 (0.25)	0.12 (0.32)	0.56 (0.50)	0.14 (0.35)	0.43 (0.49)
Public tap water	1 if public tap water source, 0 otherwise	0.07 (0.26)	0.47 (0.49)	0.12 (0.33)	0.26 (0.44)	0.27 (0.44)	0.28 (0.45)
Bore hole water	1 if bore hole water source, 0 otherwise	0.47 (0.50)	0.19 (0.39)	0.07 (0.25)	-	0.31 (0.46)	0.23 (0.42)
Protected well water	1 if protected well water source, 0 otherwise	0.18 (0.39)	0.13 (0.34)	0.25 (0.43)	0.09 (0.29)	0.04 (0.28)	0.04 (0.20)
Unprotected water	1 if unprotected well water, 0 otherwise	0.14 (0.35)	0.10 (0.25)	0.13 (0.34)	0.08 (0.28)	0.20 (0.40)	0.01 (0.11)
Covered private toilet	1 if covered private toilet, 0 otherwise	0.16 (0.37)	0.17 (0.37)	0.04 (0.19)	-	0.16 (0.37)	-
Cover shared toilet	1 if covered shared toilet, 0 otherwise	0.61 (0.49)	0.62 (0.48)	0.25 (0.43)	-	0.05 (0.22)	-
VIP latrine private toilet	1 if VIP private toilet, 0 otherwise	0.02 (0.13)	0.08 (0.26)	0.05 (0.22)	0.06 (0.23)	0.64 (0.48)	0.09 (0.29)
Pit latrine	1 if pit latrine, 0 otherwise	-	-		0.48 (0.31)		0.76 (0.42)
Flush toilet	1 if flush toilet, 0 otherwise	0.12 (0.19)			0.42 (0.38)		0.13 (0.34)
Observations		242	263	873	1363	662	1,479

Table 1 contains descriptive statistics of key variables used in the analyses. The explanatory variables included in the OLS model and the features in the ML models are selected based on their perceived relationship with the house rental price as supported by economic theory and housing economic literature (e.g. [4, 6, 47]). The average rent amount in each country does not vary significantly over the years. The rental rate per month in U.S. Dollars is around $22 for Uganda and Tanzania. There is higher monthly rent variation in Uganda (standard deviation of $36.20 in the year 2012) as compared to Tanzania (standard deviation of $21.30 in the year 2014). In Malawi, the mean monthly rental rates are $20.34 with a standard deviation of $ $23.37 in year 2014, and $17 with a standard deviation of $19.96 in year 2016. The majority of houses in all countries have roofs, floors, and external walls made up of mud. The average number of rooms per house ranged from 1.61 in Tanzania in year 2014 to 2.25 in Malawi in 2016. Percentage of households with access to electricity ranged from 37 to 59%. The types of water sources include private tap water, public tap water, borehole water, protected well water, and unprotected water. There is no consistent pattern in the type of water source used in the three countries. Public tap water in Uganda (47%) and Malawi (27%) and protected well water in Tanzania (25%) are relatively dominant sources of water used. The type of toilets available are covered private and shared toilets, VIP and uncovered latrine toilets, and flush toilets. The main type of toilet for both years in Uganda is shared toilet (61%) but it varies by year in Tanzania and Malawi. In the year 2014, VIP latrine type of toilet turned out to be the main type of toilet in Malawi.

## Results and discussions

The manuscript aims to examine multiple ML approaches (i.e. Ridge, LASSO, Tree, Random Forest, Bagging, and Boosting) and hedonic pricing approach to predict house rental values. The estimations of both the hedonic and ML models are carried out in R statistical software. The detailed R package commands for ML are presented in James et al. [41]. Model diagnostic tests of the hedonic pricing model is presented first. The determinants of house rental values are presented next, followed by in-sample and out-of-sample predictions.

### Hedonic pricing model diagnostic tests

To avoid the influence of outliers on the estimated regression coefficients, data points falling below and above three standard deviations of the mean in each dataset are removed prior to estimations. For example, the mean rental rate in Uganda in the year 2010 for the 6 observations flagged as outliers is $104 US dollars, which is higher than the mean value of $22 US dollars by more than 3 standard deviations. To test the severity of multicollinearity in the OLS regression, a Variance Inflation Factor (VIF) is used. Results show that the VIF indexes of the variables are below 4, except for the variables ‘bore hole water’ ‘public tap water’ and ‘protected well water’ in the Uganda data exceeding 5 but still less than 10. The null hypothesis of homoscedasticity (constant variance) is assessed based on the standard Breusch-Pagan test at a significance level of 5% [48]. Results showed that the null hypothesis of constant variance is not rejected for all models except in the dataset for 2014 in Tanzania and both years in Malawi. To overcome heteroscedasticity, predictions using the hedonic pricing model for the year 2014 in Tanzania and both years in Malawi are done by applying generalized weighted least squared regression methods.

Hedonic pricing models are also prone to spatial correlation [23, 24] especially when location is an important factor such as in the current study. Lack of adequate treatment of spatial dependence in the estimation of the hedonic pricing models could result in faulty results. We tested spatial autocorrelation in the model using Moran’s I test [32]. We tested the spatial dependence using univariate Moran’s I for the dependent variable, housing rental value. The spatial distribution of the households is measured using geolocation of the houses based on the longitude and latitude coordinates. We used arc distance in kilometers to measure the closeness of the households. Results indicated that we reject at the 1% significance level that the null hypothesis of no spatial dependence (significant Moran’s I) across all years and countries of study To account the presence of spatial autocorrelation[49], we implemented both the spatial lag model (SAR) the spatial error model (SEM) outlined in the methodology section [9–11].

### Determinants of house rental values

Tables 2–4 present the results from the OLS and various machine-learning approaches used in the article to estimate the determinants of house rental values in Uganda, Tanzania and Malawi. The significance of the explanatory variables in the OLS models, including the SAR and SEM specifications, are consistent in the two periods for the three countries providing robust estimates for the determinants of house rental values in these countries. We chose to include the OLS results in Tables 2–4 from the model specifications without the spatial autocorrelation only in the interest of space, although the results of the prediction performances of all specifications are presented later in Tables 9–11. District fixed effect variables are used in the estimation of the models and the specific results for these variables are not reported in the tables in the interest of space. In Uganda, factors that are significantly associated with house rental values are number of rooms, availability of electricity, floor, water characteristics and flush toilet (Table 2). In Tanzania, number of rooms, availability of electricity, water, and toilet characteristics, are significantly associated with the house rental values (Table 3). In Malawi, roof, number of rooms, electricity, toilet, and water characteristics are significantly associated with the house rental values (Table 4). None of the other explanatory variables is significantly associated with the dependent variables in any of the three countries.

**Table 2:** Determinants of housing rental values based on Ordinary Least Squares (OLS), LASSO, and ridge regressions models in Uganda.

Variables^+^	2010			2012		
	OLS	Ridge	LASSO	OLS	Ridge	LASSO
Constant	1.66	1.78	1.77	1.09 (55.60)	1.98	1.70
Dwelling	0.40 (0.18)	0.16	0.11	0.22 (0.14)	0.08	0.13
Roof	-0.38 (0.33)	0.01	-	0.23 (0.73)	-0.27	-0.09
Floor	0.64** (0.24)	0.28	0.29	0.01 (0.09)	-0.08	-
External wall	0.32 (0.22)	0.28	0.29	0.40** (0.18)	0.29	0.37
Number of rooms	0.36** (0.18)	0.22	0.12	6.43** (2.34)	0.39	0.53
Electricity	0.60*** (0.16)	0.38	0.49	0.50*** (0.11)	0.42	0.49
Private tap water	2.36*** (0.85)	0.94	1.15	0.29 (0.30)	0.31	0.20
Public tap water	-0.15 (0.41)	0.22	-	0.05 (0.24)	0.07	-
Bore hole water	-0.13 (0.30)	0.04	-	-0.58* (0.33)	-0.42	-0.56
Protected well water	0.02 (0.39)	-0.05	-	0.25 (0.26)	0.20	0.13
Unprotected water	-0.04 (0.33)	-0.01	-	-0.87** (0.36)	-0.54	-0.74
Covered private toilet	0.42 (0.79)	-0.13	-	0.63* (0.32)	0.17	0.21
Cover shared toilet	0.53 (0.75)	-0.02	-	0.40 (0.28)	0.02	0.04
VIP latrine toilet	0.22 (1.01)	0.09	-	-0.14 (0.45)	-0.34	-0.38
Flush toilet	0.62 (0.75)	0.06	-	1.29*** (0.49)	0.86	0.87
R^2^	0.75			0.41		

^+^ District fixed effect variables are used in the estimation of the models and the specific results for these variables are not reported in the Tables in the interest of space. There are 66 districts, 9 districts, and 32 districts included in the data from Uganda, Tanzania, and Malawi, respectively.

**Table 3:** Determinants of housing rental values based on Ordinary Least Squares (OLS), LASSO, and ridge regressions models in Tanzania.

	2014			2016		
Variables^+^	OLS	Ridge	LASSO	OLS	Ridge	LASSO
Constant	1.65 (0.13)	1.82	1.68	1.34*** (0.24)	1.77	1.33
Roof	-0.63 (0.11)	-0.53	-0.52	-0.73 (0.14)	-0.66	-0.72
Floor	-0.16 (0.07)	-0.21	-0.21	-0.28 (0.08)	-0.32	-0.28
External wall	0.42 (0.05)	0.40	0.44	-	0.11	-0.00
Number of rooms	0.66*** (0.05)	0.60	0.61	0.80*** (0.05)	0.72	0.80
Electricity	0.64*** (0.05)	0.62	0.70	0.54*** (0.05)	0.50	0.50
Private tap water	0.15* (0.09)	0.14	0.10	0.10*** (0.05)	0.05	-
Public tap water	-0.15 (0.11)	-0.19	-	0.31*** (0.09)	-0.15	-0.20
Protected well water	-0.27 (0.18)	-0.29	-0.13	-0.04 (0.12)	0.12	0.05
Unprotected water	-0.15 (0.12)	-0.16	-0.05	-	0.12	0.09
VIP latrine toilet	-0.18 (0.11)	-0.17	-0.04	0.89*** (0.24)	0.32	0.80
Flush toilet	0.03 (0.11)	0.05	0.01	0.72*** (0.22)	0.19	0.63
Pit latrine	0.14 (0.10)	0.15	0.10	0.49** (0.22)	-0.01	0.40
Private toilet	-0.16 (0.13)	-0.19	-0.07	-	-	-
Shared toilet	-0.31*** (0.07)	-0.30	-0.22	-	-	-
R2	0.75			0.58		

^+^District fixed effect variables are used in the estimation of the models and the specific results for these variables are not reported in the Tables in the interest of space. There are 66 districts, 9 districts, and 32 districts included in the data from Uganda, Tanzania, and Malawi, respectively.

**Table 4:** Determinants of housing rental values based on Ordinary Least Squares (OLS), LASSO, and ridge regression models in Malawi.

Variables^+^	2014			2016		
	OLS	Ridge	LASSO	OLS	Ridge	LASSO
Constant	1.29*** (0.21)	1.44	1.36	1.57** (0.25)	1.57	1.43
Dwelling	-	0.30	0.30	-0.01 (0.12)	0.06	0.01
Roof	1.11**	0.18	0.12	0.34 (0.10)	0.29	0.30
Floor	-	-0.15	-0.11	-0.15 (0.06)	-0.15	-0.14
External wall	0.14 (0.13)	0.11	-	0.17 (0.12)	0.15	0.09
Number of rooms	0.50*** (0.07)	0.42	0.42	0.41*** (0.05)	0.40	0.40
Electricity	0.82*** (0.08)	0.58	0.69	0.71*** (0.05)	0.67	0.72
Private tap water	0.58*** (0.18)	0.49	0.47	0.14*** (0.20)	0.17	0.15
Public tap water	0.24*** (0.09)	0.17	0.09	0.01 (0.20)	0.02	-
Bore hole water	-0.12 (0.19)	0.03	-	-0.22 (0.20)	-0.18	-0.18
Protected well water	0.06 (0.14)	0.05	-	-0.07 (0.22)	-0.08	-0.04
Unprotected water	0.55 (0.41)	0.41	0.09	-0.21 (0.28)	-0.17	-0.10
VIP latrine toilet	0.13 (0.13)	0.04	-	0.24 (0.15)	-0.07	-
Flush toilet	0.26 (0.17)	0.26	0.20	0.86*** (0.16)	0.52	0.61
Pit latrine	-	-0.05	-	0.14 (0.14)	-0.18	-0.07
R2	0.75			0.65		

^+^District fixed effect variables are used in the estimation of the models and the specific results for these variables are not reported in the Tables in the interest of space. There are 66 districts, 9 districts, and 32 districts included in the data from Uganda, Tanzania, and Malawi, respectively.

Of the significantly associated variables, for example, house rent increases with the increase of number of rooms. If the number of rooms is increased by one, average monthly aggregate house rent increases by about $0.36–6.43 in Uganda, $0.66–0.80 in Tanzania, and $0.41–0.50 in Malawi. The effect of electricity on house rent is also positive. Rental values of houses with access to electricity are higher by $0.50–0.60 per month in Uganda, $0.54–0.64 in Tanzania, and $0.71–0.82 in Malawi. These results imply the essence of electricity for household purposes (e.g. lighting, cooking, etc.), mechanizations (farming and non-farming), and communication and other purposes [50]. Private tap water is also positively associated with the value of house rent in all the three countries. On average, a house rent in dwellings with a private water tap are higher by $2.36 in the year 2010 in Uganda, by $0.15 in the year 2014 in Tanzania, and by $0.58 in the year 2014 in Malawi. North and Griffin [51] found that households value in-house piped water sources higher than any other house characteristics which is in agreement with the results of the current work. Rental houses increase with access to clean water because more households want to stay closer to clean water [52].

Mullainathan and Spiess [14] noted that machine learning algorithms are not built for parameter estimation and hence the estimated regression coefficients are rarely consistent. The results of the estimated coefficients from the ML approaches are estimated with this caveat in mind. However, in general, results from the Ridge ML regression models show that most of the estimated coefficients of the explanatory variables are smaller in magnitude compared to the OLS coefficients (Tables 2–4). This is consistent with the literature [41] in that Ridge regression shrinks the estimated coefficients and reduces the variance resulting in increased predictive power of the covariates. Similarly, the LASSO regression approach shrinks the variable estimates, and, in some cases, the coefficients are reduced to zero. When the estimated variable coefficients are zero, then the variables may not play a role in predicting the value of the dependent variables. In Uganda, the variable estimates that were reduced to zero under LASSO regression are roof (in the year 2010), floor (in the year 2012), public tap water, bore hole water, protected well water, covered private toilet, covered shared toilet, VIP latrine, and flush toilet (in the year 2010). In Tanzania, the estimated coefficient values of public tap water (in the year 2014) and private tap water (in the year 2016) are reduced to zero. For Malawi, the variables with zero estimated coefficients in the year 2014 under LASSO regression are external wall, borehole water, protected water well, VIP and pit latrine type of toilet. Variables that are reduced to zero in the year 2016 under LASSO estimation are public water tap, and VIP latrine type of toilet.

**Fig 2:**
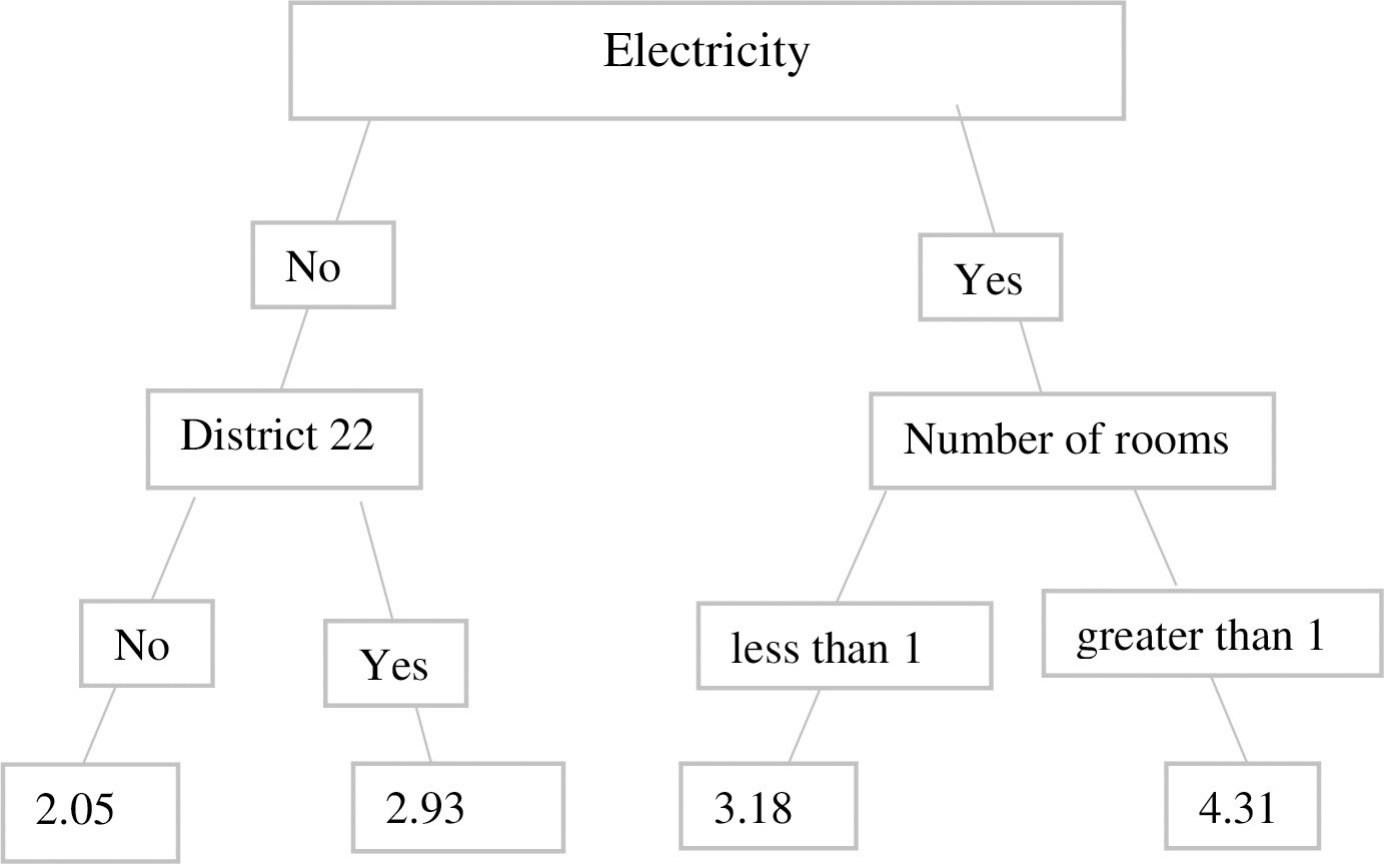
Tree regression in Uganda for 2012.

Unlike OLS, Ridge, and LASSO, the other ML approaches (i.e. the Regression Tree, Bagging, Random Forest, and Boosting) do not provide the estimated coefficients. With the Regression Tree method, the prediction of the house rental values is done by using the mean of the group at each node of the tree. For example, in Uganda, variables selected in the Regression Tree by rank are availability of electricity, and number of rooms (Fig 2). The figures of the results for the rest of the Regression Trees for other countries and years are not presented here in the interest of space. For Uganda, the variables selected in the Regression Tree in the year 2010 by rank are availability of electricity, floor, and number of rooms. Regression Tree is highly criticized in that changing the data slightly brings substantial change in tree construction [41]. The variables used in the Regression Tree in Tanzania are electricity, number of rooms, and external wall in the year 2014; and number of rooms, electricity, and dwelling in the year 2016. In Malawi, variables such as electricity, private water tap and district 16 in 2014; and electricity, flush toilet, and roof in 2016 are used in the Tree Regression.

As previously stated, the Bagging method is an extension of the Regression Tree. We construct numerous trees through resampling multiple times from the same data (i.e. bootstrapping) and then we average the entire prediction. In each Bagging, 500 trees are created using all of the variables. Results from the Bagging are presented in Tables 5–7 for the three countries. The results from the Bagging regression are ordered according to their importance in the prediction process. For example, in the year 2010 in Uganda, the four top most variables used during the Bagging regression by rank are electricity, external wall, floor and number of rooms (Table 5). The arrangement of variables varies by year and by country of analysis (see Tables A1 and A2 in S1 Appendix).

**Table 5:** List of explanatory variables by importance in predicting housing rental values using bagging regression in Uganda.

Variable^+^	Relative information	Variable	Relative information
2010		2012	
Electricity	22.68	Electricity	28.51
External wall	12.20	Number of rooms	13.34
Floor	10.98	District 20	13.09
Number of rooms	10.09	Flush toilet	7.49
Private water tap	7.09	External wall	5.56
District 20	6.67	Floor	5.32
Dwelling	6.12	Dwelling	4.83
District 64	2.86	District 64	3.55
Public tap water	2.60	Unprotected well	3.53
Flush toilet	2.31	Private water tap	3.44
Borehole water	2.14	Borehole water	2.93
District 27	2.07	Public water tap	2.71
District 33	1.79	Shared toilet	2.65
Unprotected well	1.75	Private toilet	2.01
Private toilet	1.66	Protected well	2.00
Protected well	1.66	District 41	1.90
District 32	1.54	District 37	1.74
Shared toilet	1.54	VIP toilet	1.64
District 37	1.52	District 51	1.36
Roof	1.22	District 4	1.04

^+^ District fixed effect variables are used in the estimation of the models and the specific results for these variables are not reported in the Tables in the interest of space. There are 66 districts, 9 districts, and 32 districts included in the data from Uganda, Tanzania, and Malawi, respectively.

**Table 6:** List of explanatory variables by importance in predicting housing rental values using random forest regression in Uganda.

Variable^+^	Relative information	Variable	Relative information
2010		2012	
Electricity	11.20	Electricity	7.89
External wall	8.46	District 20	4.43
Floor	6.83	Number of rooms	3.63
District 20	5.44	Flush toilet	3.08
Private tap water	3.87	Private water tap	2.63
Number of rooms	3.62	External wall	2.25
Dwelling	2.97	Floor	2.06
Public tap water	2.70	Unprotected water	1.99
Borehole water	1.93	Borehole water	1.58
Protected well	1.49	Public tap water	1.20
Private toilet	1.34	District 4	1.09
Roof	1.22	District 37	0.99
District 37	1.13	District 41	0.82
District 64	1.08	dwelling	0.72
District 37	1.00	Private toilet	0.49
District 55	0.91	Shared toilet	0.47
Flush toilet	0.79	Protected well	0.45

^+^ District fixed effect variables are used in the estimation of the models and the specific results for these variables are not reported in the Tables in the interest of space. There are 66 districts, 9 districts, and 32 districts included in the data from Uganda, Tanzania, and Malawi, respectively.

**Table 7:** List of explanatory variables by importance in predicting housing rental values using boosting regression in Uganda.

Variable^+^	Relative information	Variable	Relative information
2010		2012	
Private water tap	25.78	Electricity	24.00
Electricity	21.56	Private water	23.85
Number of rooms	18.45	Number of rooms	19.87
District 20	9.41	District 22	9.77
Public water tap	8.15	Public water tap	6.27
Protected well	5.72	Protected well	5.57
Dwelling	4.18	Dwelling	4.17
Floor	2.41	Floor	2.78
Shared toilet	1.82	External wall	1.70
External wall	1.36	Shared toilet	1.45
District 64	0.66	District 68	0.33
Private toilet	0.18	Private toilet	0.16
Borehole water	0.16	Uncovered latrine	0.05
Roof	0.09	Bore hole	0.02
Uncovered latrine	0.04	VIP shared latrine	0.01
VIP shared latrine	0.02	Unprotected well	0.01
Unprotected well	0.01	roof	0.00
VIP private latrine	0.00	VIP private latrine	0.00
Flush toilet	0.00	Flush toilet	0.00
District 1	0.00	District 1	0.00
		District 2	0.00

^+^District fixed effect variables are used in the estimation of the models and the specific results for these variables are not reported in the Tables in the interest of space. There are 66 districts, 9 districts, and 32 districts included in the data from Uganda, Tanzania, and Malawi, respectively.

Random Forest is similar to Bagging, but instead of averaging all the predictors, the approach selects some of the predictors via random selection to perform the prediction. Results of the Random Forest regressions for Uganda are shown in Table 6. The top four variables randomly selected under Random Forest in Uganda are electricity, external wall, floor and district 20 in the year 2010 (Table 6); and are electricity, district 20, number of rooms and flush type of toilet in the year 2012 (Table 6). Results of the Random Forest regressions for Tanzania (Table A3 in S1 Appendix) and Malawi (Table A4 in S1 Appendix) are reported in the S1 Appendix in the interest of space.

The results from the Boosting Regression, which is another extension to the Tree Regression, are presented in Table 7. The estimation output from the Boosting Regression are arranged in order of their importance to the prediction process. For instance, in Uganda in the year 2010, the top three variables used in Boosting by order of importance are private tap water, electricity, and number of rooms; whereas the four variables that are not used for prediction in the Boosting regression are VIP private latrine, flush toilet, and a district information (Table 7). A number of variables are not used in the Boosting Regression of different countries and in different years as shown in Tables A5 and A6 in the S1 Appendix.

### In-sample prediction

The main strength of ML approaches is their predictive capability. Machine Learning approaches outperform OLS regression, especially when the number of determinants is large [53]. The Mean Squared Error and R Squared are two of the methods used to compare the prediction performance between the ML approaches and the standard OLS regression. The Mean Squared Error is used to compare the results of the ML approaches and the standard OLS regression. The model with minimum average MSE of the predictions is considered as the best model for prediction. The MSE results for the ML approaches are presented as a proportion of the MSE of the OLS model such that the MSE of the OLS model will be equal to 1. The results of the in-sample prediction comparisons of these models is presented in Table 8.

**Table 8:** In-sample prediction performances based on standardized mean squared errors of predicting housing rental values by country and years of analysis.

	Uganda		Tanzania		Malawi		Overall performance score
	2010	2012	2014	2016	2014	2016	
OLS^+^	1.00	1.00	1.00	1.00	1.00	1.00	-
Ridge	1.03	1.01	1.01	1.01	1.01	1.01	0%
LASSO^+ +^	1.03	1.01	1.01	1.01	1.01	1.01	0%
Tree	0.95	0.98	1.06	1.59	1.13	1.07	33%
Bagging	0.94	0.93	0.97	0.68	0.91	0.98	100%
Forest	0.71	0.89	0.94	0.51	0.77	0.90	100%
Boosting	0.86	0.90	0.88	0.48	0.76	0.89	100%

^+^ OLS = Ordinary Least Squares

^+ +^LASSO = Least Absolute Shrinkage and Selection Operator.

An overall performance score is developed to rank the ML approaches in relation to the OLS approach. The score is developed by adding the number of instances the MSE in each year of analysis is lower than the MSE of the OLS model. Then, this number is divided by 6 (for a total of 6 comparisons with 3 countries and 2 years) and then multiplied by 100. The score ranges from 0 to 100% and the higher the percentage, the better the performance. A 50% threshold is used to determine whether a particular model outperformed the OLS predictions. A score of 50% indicates that the model outperformed the OLS model in 3 out of the 6 datasets, indicating that both models are equally preferred. The overall performance score is shown in the last column of Table 8. Based on this score, Boosting, Bagging, Random Forest Regression outperformed all other ML and OLS approaches for all the data sets. Ridge, LASSO and Tree Regressions have been outperformed by the OLS in-sample predictions in all countries and all years.

### Out-of-sample prediction

As previously mentioned, ML approaches are believed to provide better out-of-sample predictive values considering higher dimensionality of data structures. The results of out-of-sample prediction performance of the models are presented in Tables 9–11.

**Table 9:** Out-of-sample prediction performances based on standardized mean squared errors of predicting housing rental values by country and by year, accounting spatial lag autocorrelation (SAR).

	Uganda		Tanzania		Malawi		Overall performance score
	2010	2012	2014	2016	2014	2016	
OLS^+^	1.00	1.00	1.00	1.00	1.00	1.00	-
Ridge	0.94	0.98	0.99	0.96	0.95	1.01	83%
LASSO^+ +^	0.88	0.96	0.99	0.88	0.95	1.01	83%
Tree	0.83	0.86	1.11	1.59	1.12	1.06	33%
Bagging	0.78	0.83	0.97	1.28	0.87	0.91	83%
Forest	0.82	0.91	1.00	0.80	0.84	0.89	83%
Boosting	0.87	0.88	0.96	0.95	0.86	0.91	100%

^+^OLS = Ordinary Least Squares

^+ +^LASSO = Least Absolute Shrinkage and Selection Operator.

**Table 10:** Out-of-sample prediction performances based on standardized mean squared errors of predicting housing rental values by country and by year, accounting for spatial error autocorrelation (SEM).

	Uganda		Tanzania		Malawi		Overall performance score
	2010	2012	2014	2016	2014	2016	
OLS^+^	1.00	1.00	1.00	1.00	1.00	1.00	-
Ridge	0.93	0.92	1.01	0.89	0.96	1.01	66%
LASSO^+ +^	0.92	0.93	1.01	0.96	0.95	1.01	66%
Tree	0.88	0.86	1.13	1.30	1.13	1.05	33%
Bagging	0.86	0.76	0.99	0.89	0.88	0.92	100%
Forest	0.83	0.82	1.00	0.88	0.83	0.89	83%
Boosting	0.91	0.84	0.97	0.99	0.88	0.92	100%

^+^OLS = Ordinary Least Squares

^+ +^LASSO = Least Absolute Shrinkage and Selection Operator.

**Table 11:** Out-of-sample prediction performances based on standardized mean squared errors of predicting housing rental values by country and by year without accounting for spatial autocorrelation.

	Uganda		Tanzania		Malawi		Overall performance score
	2010	2012	2014	2016	2014	2016	
OLS^+^	1.00	1.00	1.00	1.00	1.00	1.00	-
Ridge	0.94	0.96	0.99	0.97	0.95	1.01	83%
LASSO^+ +^	0.89	0.92	1.00	0.88	0.95	1.01	83%
Tree	0.84	0.83	1.11	1.60	1.11	1.05	33%
Bagging	0.78	0.80	0.98	1.28	0.88	0.91	83%
Forest	0.82	0.88	1.00	0.81	0.84	0.88	83%
Boosting	0.88	0.85	0.96	0.96	0.87	0.91	100%

^+^OLS = Ordinary Least Squares

^+ +^LASSO = Least Absolute Shrinkage and Selection Operator.

Tables 9 and 10 contain results of the spatial lag model (SAR) and the spatial error model (SEM) respectively. These tables present the standardized MSE values of the OLS and the ML approaches. Overall, except Tree Regression, all ML methods are preferred to the OLS model in predicting house rental values using an out-of-sample data set for all of the countries and years. Boosting Regression is superior to OLS in terms of out-of-sample predictions in all of the datasets. Ridge, LASSO, Bagging, and Forest Regression outperformed OLS in most of the datasets.

An overall performance score is also developed for the out-of-sample predictions. The results of such overall performance score comparisons are presented in the final columns of Tables 9–11. Based on this score, Boosting is the top performer in terms of out-of-sample predictions, followed by Bagging, Random Forest and Ridge and LASSO Regressions. Unlike in the in-sample predictions, Ridge and LASSO regressions performed better than OLS in the out-of-sample predictions. Tree Regression is again the least performer in the group.

## Conclusion and recommendation

Housing value is a major component of the household’s aggregate expenditure, a commonly used metric for welfare analysis. Data shows that a significant proportion of households in a typical household survey, adopted by the Word Bank are self-owned, and predicting the true market price of housing service are crucial to obtain true assessment of economic well-being. Several Machine Learning approaches (Ridge, LASSO, Tree, Random Forest, Bagging, and Boosting), in reference to hedonic pricing approach, are evaluated in this article for the purpose of predicting the rental values of housing in household level survey data from three sub-Saharan countries (Uganda, Tanzania, and Malawi).

Results from the OLS specifications and various machine-learning approaches used to estimate the determinants of house rental values in Uganda, Tanzania, and Malawi are presented. The significance of the explanatory variables in the OLS models are consistent across all the data sets providing robust estimates for the determinants of house rental values in these countries. Most variables selected by all the ML approaches are similar with what the OLS regression identified as determinants of rental values: the number of rooms, availability of electricity, private tap water, and toilet characteristics.

The Mean Squared Error (MSE) is used to compare the prediction performance of the ML approaches and the OLS regressions. The model with minimum average MSE of the predictions is considered as the best model for prediction. In general, Boosting is the best model in predicting house values using out-of-sample data set for all the countries and all the years. Tree is the least performer in the group. Although hedonic pricing models using OLS regression provide consistent and reliable predictions of house rental values, especially when the statistically properties (e.g. consistency and unbiasedness of estimated coefficients) of the explanatory variables are of interest, most of the ML approaches tend to provide alternative predictive performances, both in in-sample and out-of-sample datasets. Therefore, while predicting the rental value non-rented houses in household surveys, the ML methods could be used as alternative approaches. With the availability of abundant data and better computing power, ML methods provide viable alternative to predicting housing values in household surveys. Finally, whether a particular ML model is preferred to hedonic pricing model for predicting rental value of housing is driven by the particular dataset, and one has to empirically test among the range of available methods.

The current work could be extended by including data set from multiple countries and across time. Future work should also include evaluations of consumption aggregates and welfare comparisons using house values estimated by the hedonic pricing and ML approaches.

## Supporting information

S1 Appendix (DOCX)
