# Supplementary material for: Predicting the rental value of houses in household surveys in Tanzania, Uganda and Malawi: Evaluations of hedonic pricing and machine learning approaches
Source: PLoS One. 2021 Feb 11;16(2):e0244953. doi: 10.1371/journal.pone.0244953 (PMC7877618; doi:10.1371/journal.pone.0244953)
Supplement: S1 Appendix — (DOCX) [file pone.0244953.s001.docx]

**Appendix**:

**Table A1. List of Explanatory Variables by Importance in Predicting Housing Rental Values Using Bagging Regression in Tanzania**

| Variable^+^ | Relative information | Variable | Relative information |
| --- | --- | --- | --- |
| 2014 | | 2016 | |
| Electricity | 115.84 | Electricity | 57.91 |
| Number of rooms | 57.42 | Number of rooms | 44.71 |
| External wall | 47.62 | Floor | 22.79 |
| Floor | 33.82 | Dwelling | 20.28 |
| Shared toilet | 23.23 | External wall | 15.03 |
| Roof | 11.48 | District 10 | 13.34 |
| District 1 | 7.49 | Pit latrine | 7.60 |
| Private tap water | 6.46 | Roof | 6.35 |
| District 3 | 4.28 | Public tap water | 6.06 |
| VIP private toilet | 4.14 | Flush toilet | 5.68 |
| Flush toilet | 4.09 | Private water tap | 3.86 |
| District 2 | 4.05 | District 5 | 3.86 |
| District 4 | 3.92 | VIP toilet | 2.62 |
| Uncovered latrine | 3.87 | District 3 | 2.60 |
| District 5 | 3.87 | Unprotected water well | 2.38 |
| VIP shared toilet | 3.81 | Protected water well | 1.87 |
| District 6 | 3.81 | District 12 | 1.74 |
| Public tap water | 3.60 | District 16 | 1.34 |
| Private toilet | 3.27 | District 1 | 1.23 |
| Protected well | 3.01 | District 11 | 1.10 |
| District 7 | 3.01 | District 14 | 1.05 |

^+^ District fixed effect variables are used in the estimation of the models and the specific results for these variables are not reported in the Tables in the interest of space. There are 66 districts, 9 districts, and 32 districts included in the data from Uganda, Tanzania, and Malawi, respectively.

**Table A2. List of Explanatory Variables by Importance in Predicting Housing Rental Values Using Bagging Regression in Malawi**

| Variable^+^ | Relative information | Variable | Relative information |
| --- | --- | --- | --- |
| 2014 | | 2016 | |
| Electricity | 72.88 | Electricity | 172.81 |
| Number of rooms | 26.78 | Private water tap | 54.82 |
| Private water tap | 21.82 | Flush toilet | 51.52 |
| District 16 | 19.19 | Number of rooms | 46.60 |
| Flush toilet | 17.39 | District 17 | 33.15 |
| Public water tap | 12.38 | Floor | 32.85 |
| Dwelling | 11.99 | District 7 | 27.24 |
| Floor | 11.25 | Roof | 25.78 |
| District 31 | 7.66 | Borehole water | 19.70 |
| Roof | 7.31 | Pit latrine | 15.20 |
| Traditional latrine | 3.54 | Dwelling | 8.78 |
| District 30 | 3.06 | District 30 | 6.70 |
| Unprotected well | 2.98 | Unprotected well | 6.37 |
| District 7 | 2.69 | District 7 | 5.55 |
| District 27 | 2.51 | District 27 | 4.72 |
| District 19 | 2.30 | District 19 | 4.59 |
| District 26 | 2.24 | District 16 | 3.79 |
| Latrine without roof | 2.19 | District 12 | 3.72 |
| External wall | 1.70 | District 4 | 3.20 |

^+^ District fixed effect variables are used in the estimation of the models and the specific results for these variables are not reported in the Tables in the interest of space. There are 66 districts, 9 districts, and 32 districts included in the data from Uganda, Tanzania, and Malawi, respectively.

**Table A3. List of Explanatory Variables by Importance in Predicting Housing Rental Values Using Random Forest Regression in Tanzania**

| Variable^+^ | Relative information | Variable | Relative information |
| --- | --- | --- | --- |
| 2014 | | 2016 | |
| Electricity | 84.50 | Electricity | 38.85 |
| Number of rooms | 43.71 | Number of rooms | 26.31 |
| External wall | 42.03 | Floor | 18.65 |
| Floor | 35.03 | Dwelling | 16.11 |
| Shared toilet | 27.22 | External wall | 14.71 |
| Roof | 13.39 | District 7 | 11.89 |
| Private tap water | 7.60 | Pit latrine | 7.17 |
| District 1 | 6.31 | Roof | 6.88 |
| Unprotected well | 5.14 | Public tap water | 5.56 |
| Flush toilet | 4.74 | Private tap water | 3.63 |
| Private toilet | 4.74 | District 5 | 3.08 |
| VIP private toilet | 3.66 | VIP toilet | 2.13 |
| District 2 | 3.43 | District 2 | 1.99 |
| District 6 | 3.20 | Protected well | 1.84 |
| Public tap water | 3.12 | Unprotected well | 1.55 |
| VIP public toilet | 3.06 | District 17 | 1.37 |
| District | 3.05 | District 16 | 1.33 |
|  |  | District 24 | 1.08 |

^+^ District fixed effect variables are used in the estimation of the models and the specific results for these variables are not reported in the Tables in the interest of space. There are 66 districts, 9 districts, and 32 districts included in the data from Uganda, Tanzania, and Malawi, respectively.

**Table A4. List of Explanatory Variables by Importance in Predicting Housing Rental Values Using Random Forest Regression in Malawi**

| Variable^+^ | Relative information | Variable | Relative information |
| --- | --- | --- | --- |
| 2014 | | 2016 | |
| Electricity | 42.28 | Electricity | 100.22 |
| Private tap water | 18.67 | Flush toilet | 40.91 |
| Number of rooms | 17.72 | Private tap water | 40.57 |
| Flush toilet | 16.92 | Floor | 32.90 |
| District 16 | 11.97 | Roof | 23.17 |
| Floor | 11.14 | Borehole water | 22.64 |
| Dwelling | 10.30 | Number of rooms | 22.10 |
| Roof | 9.45 | Pit latrine | 19.84 |
| Public tap water | 6.52 | Dwelling | 14.52 |
| Traditional latrine | 4.54 | District 17 | 13.80 |
| District 31 | 3.01 | District 7 | 8.89 |
| District 27 | 2.64 | District 1 | 4.83 |
| Latrine without roof | 2.46 | Public tap water | 4.51 |
| District 5 | 2.04 | VIP toilet | 3.36 |
| District 7 | 1.97 | District 2 | 3.36 |
| District 30 | 1.69 | Protected well | 2.79 |
| Unprotected well | 1.54 | District 13 | 2.79 |
| External wall | 1.37 | District 12 | 2.59 |

^+^ District fixed effect variables are used in the estimation of the models and the specific results for these variables are not reported in the Tables in the interest of space. There are 66 districts, 9 districts, and 32 districts included in the data from Uganda, Tanzania, and Malawi, respectively.

**Table A5. List of Explanatory Variables by Importance in Predicting Housing Rental Values Using Boosting Regression in Tanzania**

| Variable^+^ | Relative information | Variable | Relative information |
| --- | --- | --- | --- |
| 2014 | | 2016 | |
| Number of rooms | 33.59 | Number of rooms | 45.82 |
| Private water tap | 25.29 | Electricity | 14.60 |
| District 1 | 12.60 | External wall | 10.53 |
| roof | 10.62 | Flush toilet | 9.10 |
| Electricity | 7.57 | District 1 | 8.91 |
| VIP private latrine | 3.94 | Private water tape | 8.20 |
| External wall | 3.44 | Uncovered latrine | 2.63 |
| Uncovered latrine | 1.37 | Public tap water | 0.07 |
| Shared toilet | 1.22 | District 3 | 0.04 |
| floor | 0.16 | District 2 | 0.03 |
| VIP shared latrine | 0.13 | District 5 | 0.03 |
| Unprotected well | 0.04 | District 8 | 0.01 |
| Public water tap | 0.00 | floor | 0.01 |
| District 2 | 0.09 | VIP share latrine | 0.00 |
| District 8 | 0.04 | Shared toilet | 0.00 |
| District 3 | 0.02 | Private toilet | 0.00 |
| District 4 | 0.01 | VIP private latrine | 0.00 |
| District 5 | 0.00 | - | - |
| District 6 | 0.00 | - | - |
| District 7 | 0.00 | - | - |
| Borehole water | 0.00 | - | - |

^+^ District fixed effect variables are used in the estimation of the models and the specific results for these variables are not reported in the Tables in the interest of space. There are 66 districts, 9 districts, and 32 districts included in the data from Uganda, Tanzania, and Malawi, respectively.

**Table A6. List of Explanatory Variables by Importance in Predicting Housing Rental Values Using Boosting Regression in Malawi**

| Variable^+^ | Relative information | Variable | | Relative information |  |
| --- | --- | --- | --- | --- | --- |
| 2014 | | | 2016 | | |
| Private water tap | 38.12 | Number of rooms | | 29.43 |  |
| Number of rooms | 30.32 | Private water tap | | 27.31 |  |
| District 31 | 13.00 | Flush toilet | | 21.42 |  |
| District 16 | 10.00 | District 6 | | 15.19 |  |
| Electricity | 7.42 | Electricity | | 3.80 |  |
| District 6 | 5.56 | Traditional latrine | | 1.06 |  |
| Public water tap | 4.19 | District 26 | | 0.66 |  |
| Floor | 1.91 | District 31 | | 0.48 |  |
| External wall | 1.49 | Public water tap | | 0.30 |  |
| District 30 | 0.01 | District 30 | | 0.16 |  |
| Roof | 0.01 | roof | | 0.13 |  |
| District 20 | 0.01 | External wall | | 0.04 |  |
| Dwelling | 0.00 | floor | | 0.01 |  |
| District 4 | 0.00 | Dwelling | | 0.00 |  |
| District 10 | 0.00 | Unprotected water | | 0.00 |  |
| VIP latrine | 0.00 | borehole | | 0.00 |  |
| - | - | Protected water | | 0.00 |  |

^+^ District fixed effect variables are used in the estimation of the models and the specific results for these variables are not reported in the Tables in the interest of space. There are 66 districts, 9 districts, and 32 districts included in the data from Uganda, Tanzania, and Malawi, respectively.
